# Randomized Controlled Trial of a Group Cognitive Behavior Therapy HIV Prevention Intervention for Immigrant Latino Gay, Bisexual, and Other Men who Have Sex with Men

**DOI:** 10.1007/s10461-025-04906-5

**Published:** 2025-10-14

**Authors:** Laura M. Bogart, Joanna L. Barreras, Jesse Robledo, Laura Whitaker, Alexandra Mendoza-Graf, David J. Klein, Gabriela Castro, Zachary Wagner, Daniel Penoyer, Sarah MacCarthy, Ronald A. Brooks, David W. Pantalone

**Affiliations:** 1 RAND, 1776 Main Street, P.O. Box 2138, Santa Monica, CA 90407-2138 USA; 2 Charles R. Drew University of Medicine and Science, Los Angeles, CA USA; 3 Bienestar Human Services, Inc, Los Angeles, CA USA; 4 California State University Long Beach, Long Beach, CA USA; 5 University of Southern California, Los Angeles, CA USA; 6 University of Alabama Birmingham, Birmingham, AL USA; 7 University of California Los Angeles, Los Angeles, CA USA; 8 University of Massachusetts Boston, Boston, MA USA; 9 The Fenway Institute, Fenway Health, Boston, MA USA; 10 Lehman College, City University of New York, Bronx, NY USA

**Keywords:** HIV, Stigma, Latino, Randomized controlled trial, Sexual minority men, Spanish-speaking

## Abstract

Latino gay, bisexual, and other men who have sex with men (LGBMSM) are disproportionally affected by HIV in the U.S. We tested a culturally tailored community-based group HIV prevention intervention (“*Siempre Seguiré*”) for immigrant LGBMSM that uses cognitive behavior therapy to address coping with intersectional stigma and medical mistrust. A total of 289 immigrant LGBMSM were randomized (144 control, 145 intervention) and completed surveys at baseline and 4-, 8-, and 12-months (79% retained). Intention-to-treat repeated-measures regressions indicated significant intervention effects on higher HIV testing and reduced internalized Latino and sexual minority stigma, anticipated HIV stigma, stigmatizing PrEP beliefs, and medical mistrust. Intervention cost-effectiveness was $876 per 10% increase in HIV testing per participant, which improved to $600 excluding fidelity and recruitment costs (which would likely be minimal for implementation). To expedite efforts to end the HIV epidemic, research is needed to determine strategies to implement, disseminate, and sustain *Siempre Seguiré*.

## Resumen

Los hombres latinos gay, bisexuales y otros hombres que tienen sexo con hombres (LGBMSM, por sus siglas en inglés) se ven afectados de manera desproporcionada por el VIH en los Estados Unidos. Probamos una intervención comunitaria culturalmente adaptada para la prevención del VIH («Siempre Seguiré»), dirigida a inmigrantes LGBMSM, que utiliza terapia cognitivo-conductual para desarrollar estrategias de afrontamiento frente al estigma interseccional y la desconfianza médica. Un total de 289 inmigrantes LGBMSM fueron asignados aleatoriamente (144 al grupo control y 145 al grupo intervención), y completaron encuestas al inicio y a los 4, 8 y 12 meses (79% de retención). Los análisis de regresión con medidas repetidas por intención de tratar mostraron efectos significativos de la intervención, incluyendo un aumento en la realización de pruebas de VIH y una reducción del estigma internalizado relacionado con ser latino y pertenecer a una minoría sexual, del estigma anticipado relacionado con el VIH, de las creencias estigmatizantes sobre la PrEP, y de la desconfianza médica. La intervención tuvo una rentabilidad de $876 por cada incremento del 10% en la realización de pruebas de VIH por participante, la cual mejoró a $600 al excluir los costos de fidelidad y reclutamiento (que probablemente serían mínimos en la implementación). Para acelerar los esfuerzos hacia la erradicación de la epidemia del VIH, se requieren investigaciones que determinen estrategias para implementar, difundir y sostener Siempre Seguiré.

## Introduction

Latino gay, bisexual, and other men who have sex with men (LGBMSM) in the U.S. are highly affected by HIV [1, 2]. In 2022, LGBMSM accounted for 36% of HIV diagnoses among all men who have sex with men [3]. Moreover, national data from 2022 showed that Latino/a/x individuals represented 17% of PrEP users [4, 5]. Although increased access to HIV prevention services is critical for reducing HIV rates, only one evidenced-based intervention, in which Spanish-speaking LGBMSM peer navigators educate social network members about HIV prevention, has shown effects on HIV testing in this group [6].

Intersectional stigma, including overlapping forms of oppression experienced by minoritized groups [7], contributes to suboptimal HIV outcomes. LGBMSM face multiple forms of discrimination related to their ethnicity and sexual minority identity, which can affect their mental and physical health and healthcare behaviors [8–11]. Immigration introduces an additional intersectionality, through challenges to healthcare access related to discrimination, language barriers, and potentially, undocumented immigration status and poverty [1, 12]. However, few HIV prevention interventions have addressed and shown effects on intersectional stigma directly [13].

Cognitive behavior therapy (CBT) interventions have been effective in improving stress management, as well as reducing internalized stigma, including among people with mental health conditions, people living with HIV, and gay and bisexual and other men who have sex with men [14–16]. Using community-engaged research methods [17], we developed and conducted three pilot tests of a group CBT intervention that encourages adaptive coping with intersectional stigma [18–20]. *Siempre Seguiré*, for LGBMSM, was developed through qualitative research and two pilot tests and showed effects on decreased negative emotional coping responses to discrimination, including shame (a form of internalized stigma), in a non-randomized pilot—and improved HIV treatment adherence and reduced medical mistrust in a randomized pilot [18, 20]. *Still Climbin*, for Black gay, bisexual, and other men who have sex with men, showed effects on improved adaptive coping with discrimination in a randomized pilot [19].

Based on this foundational work, we adapted *Siempre Seguiré* for the present study to focus on HIV prevention among immigrant LGBMSM of HIV-negative or unknown serostatus, and conducted a randomized controlled trial (RCT). We hypothesized that intervention participants would demonstrate a greater likelihood of HIV testing and PrEP use at follow-up compared to control participants. We also hypothesized that intervention participants would show improved coping responses to discrimination, including reduced internalized stigma (related to negative Latino, sexual minority, and HIV stereotypes), and decreased stigmatizing beliefs about PrEP and medical mistrust.

## Methods

### Study Design and Procedures

We tested the *Siempre Seguiré* intervention in an RCT in which intervention and control immigrant LGBMSM participants were enrolled in 13 consecutive cohorts and, thus, intervention participants were clustered into 13 groups for analysis [21]. Participants were randomly assigned to the intervention or control group after they completed the baseline assessment. Participants completed 90-minute surveys at baseline and 4-, 8-, and 12-month follow-up, and 10-minute check-in calls at 2-, 6- and 10-months post-baseline. Survey items were translated and back-translated by multiple native Spanish speakers on the study team, who discussed and iterated on item wording and translations together. Gift card incentives included $50 for each survey, $10 for each check-in call, and $15 for each intervention session. All intervention sessions were conducted in Spanish, and participants could complete the survey in Spanish or English. Survey data were collected between February 2021 and November 2024, and intervention sessions were delivered between April, 2021 and January, 2024.

The study was conducted under a partnership among academic researchers and community experts at Bienestar Human Services, Inc. (“Bienestar”), a community-based social services organization in Los Angeles County focused on health issues faced by Latino/a/x sexual and gender minority populations. Bienestar offers services for HIV prevention, STI testing and treatment, and mental health and substance use. Many of Bienestar’s clients are immigrants from Mexico, as well as countries in Central and South America. The study was co-designed with Bienestar research staff, who helped to develop and review all study materials. Data collection was conducted by Bienestar research staff, and Bienestar behavioral health staff and research staff facilitated intervention sessions.

The research was approved by the RAND Human Subjects Protection Committee (2024-N0028). A Data Safety and Monitoring Board (DSMB) reviewed study recruitment and retention annually and reportable events as needed. Participants provided verbal informed consent (over the phone or online) and written HIPAA consent for release of medical records (via US mail, in person, or electronically). We originally intended to conduct informed consent, data collection, and intervention sessions entirely in person, but most study procedures were moved to phone or online platforms during the COVID-19 pandemic. Some recruitment activities and intervention groups were conducted in-person using COVID-19 safety protocols.

## Intervention Description

*Siempre Seguiré* was culturally tailored with relevant content and examples on intersectional stigma from ethnicity, sexual minority identity, and immigration status. The name (translated as “I will always continue”) was drawn from a line in a well-known song associated with gay empowerment (“¿A Quién Le Importa?,” translated as “Who Cares?”). Cultural tailoring was accomplished through synthesis of the team’s expertise, reliance on the published literature [22], and consultation with Bienestar’s staff and community advisory board.

Across eight sessions, facilitators used CBT strategies to guide groups of clients through a behavioral analysis of their cognitive, affective, and behavioral responses to intersectional stigma and discrimination, discussing adaptive ways to cope while maintaining life values and goals. Session topics included: psychoeducation about intersectional stigma, discrimination, multiple identities exploration (e.g., self-description of different social identities, discussion of masculinity in relation to identity), and intersectional stigma’s effects on health and HIV prevention; using the CBT model to understand and overcome barriers to coping with intersectional stigma (e.g., evaluating effectiveness of coping strategies in relation to goals); exploring vulnerability and resilience; understanding and addressing medical mistrust and internalized stigma as ineffective coping responses; leveraging social support against stigma, and brainstorming ways to address structural stigma in one’s community. Discussions about HIV prevention, including HIV testing and PrEP, were integrated throughout the sessions (e.g., with illustrative examples), and Bienestar’s PrEP navigator visited each group twice to discuss PrEP and answer questions. Sessions began and ended with short mindfulness exercises. Participants were encouraged to complete take-home activities in which they practiced using skills discussed in each session (e.g., tracking discrimination and coping responses) and then reported back to the group. In the final session, participants received a graduation certificate during a ceremony in which facilitators commented on each participant’s strengths and growth throughout the sessions. Comprehensive descriptions of the intervention and pilot results are available in prior publications [18–20].

Of the 13 intervention groups, four were conducted in person and nine online. Online intervention sessions were adapted to be shorter and more frequent, with graphics (e.g., illustrative slides), to enhance engagement. A total of 14 online meetings were conducted over 8 weeks to cover the content of the 8 original sessions, with some sessions split over two online meetings conducted within the same week.

Three clinically trained facilitators (MSWs) and three peer co-facilitators were trained by two clinically trained Co-Investigators (DWP, JLB), one of whom (JLB) provided weekly individual supervision after listening to session recordings. Each intervention group was led by one clinically trained Latino/a facilitator and one peer LGBMSM co-facilitator. Across two team members (JLB, AMG), approximately 24% (*n* = 25 of 104) of intervention sessions were coded for fidelity to intervention content and structure fidelity; 76% of ratings were coded as “completely” covered.

## Participant Recruitment

Participants were eligible if they were aged 18 or older; were a cisgender man (assigned male at birth and currently identify as male); identified as Latino; immigrated to the U.S. from another country or migrated to the continental U.S. from Puerto Rico; reported having sex with men in the past 12 months; reported HIV-negative or unknown serostatus; anticipated being available for the next 12 months to attend study visits; and were able to communicate in spoken Spanish or English. Participants were recruited across Southern California using a range of strategies: in-person in-reach at the partner organization (e.g., client/staff presentations); in-person outreach at relevant events (e.g., Pride celebrations) and businesses (e.g., Latino/a/x LGBT-focused clubs/bars); passive recruitment via hard copy fliers left at the partner community-based organization and other relevant local organizations; social media recruitment via paid advertisements, video posts, and direct messaging (e.g., of people who “liked” study posts on Facebook/Instagram). Participants and committee advisory board members from the partner organization were offered $10 per referral, for up to three referrals.

## Primary PrEP and HIV Testing Outcomes

At all surveys and check-in time-points, participants were asked the date of their last HIV test, as well as if they were taking PrEP or had a current PrEP prescription. Self-reported HIV testing was validated using medical records data for 26% of participants reporting HIV testing at baseline (30 of 114) and 42% at follow-up (88 of 210). Participants who endorsed taking PrEP were asked the date of their last PrEP prescription (to confirm that they had received a PrEP prescription within the past 3 months). Current PrEP prescription was validated with objective data sent by health care providers from medical records, via photo-verification (of medication bottle or healthcare portal) sent by participants securely via WhatsApp©, or with urinalysis. Specifically, participants who reported current PrEP use were asked to provide a urine sample to measure adherence (i.e., protective tenofovir levels of at least 4 doses in the past 7 days) [23, 24]. Urine samples were originally sent to Synergy Medical Laboratories for analysis; however, this service was discontinued during the COVID-19 pandemic. Instead, participants were asked to conduct a self-test with a point-of-care stick and photograph the results to send to study staff. Because the pandemic presented challenges in obtaining in-person and mailed tests, urinalysis was only obtained for 88 participants at baseline or follow-up; thus, adherence was not included as an outcome and urinalysis results were only used to confirm current PrEP use. We validated self-reported PrEP use with objective data for a subset of 45% of participants (*n* = 33 of 74) at baseline and 57% (*n* = 72 of 127) for the follow-up period.

## Secondary Psychosocial Outcomes

On the baseline survey and all follow-up surveys, participants completed validated scales assessing internalized sexual minority stigma (Internalized Homophobia scale [25]; alpha = 0.90), internalized Latino stigma (with 6 items adapted from the Multidimensional Model of Racial Identity Private Regard subscale [26] and 2 items adapted from the Cross-Racial Identity Scale Self-Hatred subscale [27]; alpha = 0.67), internalized HIV stigma (adapted to assess anticipated HIV stigma from the Internalized AIDS Related Stigma Scale [28]; alpha = 0.87), and stigmatizing beliefs about PrEP (PrEP Stigma scale [29]; alpha = 0.76). Participants also completed medical mistrust scales: general medical mistrust [30] (alpha = 0.66), and a PrEP conspiracy beliefs scale (adapted from prior research [31, 32]; alpha = 0.69). All stigma and mistrust items used a 5-point Likert scale (strongly agree to strongly disagree) with a middle (neutral) option, with the exception of the internalized homophobia items, which used a 4-point scale without a middle option.

Participants completed the Brief COPE [33], a general measure of coping strategies, which, consistent with intervention goals, was adapted to assess frequency of use of each strategy for coping with discrimination, using the instructions, “indicate the extent you do what the item says when you are faced with discrimination,” and response options 1 = “I haven’t been doing this at all,” 2 = “I’ve been doing this a little bit,” 3 = “I’ve been doing this a medium amount” and 4 = “I’ve been doing this a lot.” Following guidance to derive Brief COPE subscales based on the study goals and participant population [33, 34], we conducted an exploratory factor analysis of the items with varimax rotation; we removed items with factor loadings < 0.40 and/or that were conceptually inconsistent with the factor. We identified three subscales: social support strategies (4 items, e.g., “I get emotional support from others”; alpha = 0.79); cognitive strategies (6 items, e.g., “I look for something good in what is happening”; alpha = 0.85); and negative (internalizing) strategies (4 items; “I’ve given up trying to cope”; alpha = 0.70). The mean of each psychosocial scale was used in analyses.

To assess PrEP readiness, participants were asked if they believed that they were an appropriate candidate for PrEP (definitely not, probably not, not sure, probably, definitely), and if they have or knew a medical provider who would be willing to prescribe PrEP (definitely not, probably not, don’t know, probably, definitely) [35]; these outcomes were dichotomized (0 = definitely not, probably not, or don’t know; 1 = probably or definitely).

### Potential Socio-Demographic Covariates/Moderators

Participants were asked to report their age, country of origin, immigration status, length of time living in the U.S., education level, sexual identity, employment status, and annual income (see Table 1).

**Table 1:** Baseline characteristics of *Siempre Seguiré* analytic sample by condition

	Intervention (*n* = 145) M (SD)/*n* (%)	Control (*n* = 144) M (SD)/*n* (%)	Overall (*n* = 289) M (SD)/*n* (%)	*P*-value (intervention vs. control at baseline)
**Age M (SD)**	38.3 (9.8)	37.5 (10.0)	37.9 (9.9)	0.49
**Country of Origin (%**,** n = 289)**				0.13
Mexico	97 (66.9%)	81 (56.3%)	178 (61.6%)	
Guatemala	13 (9.0%)	24 (16.7%)	37 (12.8%)	
El Salvador	17 (11.7%)	15 (10.4%)	32 (11.1%)	
Other	18 (12.4%)	24 (16.7%)	42 (14.5%)	
**Immigration Status (%**,** n = 285)**				0.04
Undocumented	66 (45.5%)	80 (57.1%)	146 (51.2%)	
US citizen	23 (15.9%)	20 (14.3%)	43 (15.1%)	
Permanent resident	24 (16.7%)	19 (13.6%)	43 (15.1%)	
Temporary visa	11 (7.6%)	6 (4.3%)	17 (6.0%)	
Asylum/refugee	12 (8.3%)	8 (5.7%)	20 (7.0%)	
Deferred Action for Childhood Arrivals (DACA)	7 (4.8%)	3 (2.1%)	10 (3.5%)	
Other	2 (1.4%)	4 (2.9%)	6 (2.1%)	
**Length of Time in U.S. (%**,** n = 289)**				0.03
Less than 1 year	9 (6.2%)	10 (6.9%)	19 (6.6%)	
1–5 years	27 (18.6%)	41 (28.5%)	68 (23.5%)	
6–10 years	14 (9.7%)	21 (14.6%)	35 (12.1%)	
11–15 years	11 (7.6%)	10 (6.9%)	21 (7.3%)	
16–20 years	33 (22.8%)	18 (12.5%)	51 (17.7%)	
More than 20 years	51 (35.2%)	44 (30.6%)	95 (32.9%)	
**Education Level (%**,** n = 289)**				0.20
None or less than high school	36 (24.8%)	26 (18.1%)	62 (21.5%)	
High school diploma/equivalent	38 (26.2%)	43 (29.9%)	81 (28.0%)	
Some college (without degree)	15 (10.3%)	15 (10.4%)	30 (10.4%)	
Associate/technical school degree or certificate	14 (9.7%)	16 (11.1%)	30 (10.4%)	
4-year college graduate	37 (25.5%)	35 (24.3%)	72 (24.9%)	
Graduate school degree	5 (3.5%)	9 (6.3%)	14 (4.8%)	
**Employed (%**,** n = 289)**				0.26
Working full-time	96 (66.2%)	85 (59.0%)	181 (62.6%)	
Working part-time	30 (20.7%)	33 (22.9%)	63 (21.8%)	
Not working: Disabled, retired, or student	5 (3.5%)	5 (3.5%)	10 (3.5%)	
Not working: other reasons	14 (9.7%)	21 (14.6%)	35 (12.1%)	
**Annual Income (%**,** n = 207)**				0.73
$0-$12,000	21 (14.5%)	19 (13.2%)	40 (13.8%)	
$12,000-$24,999	11 (7.6%)	20 (13.9%)	31 (10.7%)	
$25,000-$34,999	17 (11.7%)	12 (4.3%)	29 (10.0%)	
$35,000-$49,999	15 (10.3%)	17 (11.8%)	32 (11.1%)	
$50,000 or greater	39 (26.9%)	36 (25.0%)	75 (26.0%)	
Don’t know	40 (27.6%)	37 (25.7%)	77 (26.6%)	
Refuse	2 (1.4%)	3 (2.1%)	5 (1.7%)	
**Sexual Orientation (%**,** n = 289)**				0.20
Gay	119 (82.1%)	109 (75.7%)	228 (78.9%)	
Queer, bisexual, pansexual, or other non-monosexual	24 (16.7%)	32 (22.2%)	56 (19.4%)	
Not sure, in transition, or something else	2 (1.4%)	2 (1.4%)	4 (1.4%)	
Heterosexual	0 (0.0%)	1 (0.7%)	1 (0.4%)	
**Primary Outcomes at Baseline**				
**HIV Test**,** last 12 months (%**,** n = 282)**	86 (61.0%)	81 (57.5%)	167 (59.2%)	0.63
**Current PrEP Use (%**,** n = 289)**	40 (27.6%)	43 (29.9%)	83 (28.7%)	0.70

Note: Dichotomization for analysis: immigration status as undocumented vs. other, education as less than high school graduate vs. high school or more, employment as full-time or part-time vs. not working; annual income at <$12,000 vs. $12,000 or greater; and sexual orientation as gay vs. other identities

### Statistical Analysis

Intention-to-treat analyses [36] were used to test intervention efficacy. For HIV testing and PrEP use, we conducted logistic regressions predicting whether participants were tested for HIV or on PrEP at any point within the follow-up period (yes/no). In a post-hoc sensitivity logistic regression analysis, the HIV testing outcome was further restricted to participants who were not taking PrEP at baseline, because PrEP use requires regular HIV testing (and thus is confounded with the HIV testing outcome). For self-reported secondary psychosocial outcomes, we analyzed all follow-up responses with repeated-measures regressions which included fixed effects for intervention condition, baseline value of the outcome, and covariates, and employing a sandwich estimator to account for within-participant clustering; each participant could contribute up to three follow-up responses (from the 4-, 8-, or 12-month surveys). Linear regression was used for continuous psychosocial outcomes (stigma, coping, medical mistrust) and logistic regression was used for dichotomous psychosocial outcomes (PrEP readiness).

To address potential bias from differential randomization and drop-out, we used covariates and non-response weights. Socio-demographic characteristics that significantly differed by intervention condition at baseline (*p* <.05; immigration status and length of time in U.S.) were included as covariates. Comparisons of baseline socio-demographic characteristics were based on t-tests for continuous characteristics, Fisher’s Exact tests for binary characteristics, and chi-square tests for categorical characteristics. Nonresponse weights [37, 38] were used to address any potential differences between participants with complete data for at least one follow-up wave versus participants with only baseline data. For survey data, weights were created using the inverse of the estimated probability of completing any follow-up survey, based on a multivariable logistic regression using socio-demographic and psychosocial variables that were not missing for any participant. Participants were more likely to have completed any follow-up survey if they were older [OR (95%CI) = 1.1 per year (1.0–1.1.0.1), *p* =.02], of undocumented immigration status [OR (95% CI) = 2.9 (1.4–6.3), *p* =.006], or had at least a high school diploma [OR (95% CI) = 2.6 (1.2–5.7), *p* =.02].

Follow-up regression analyses explored within-intervention group dosage effects [39] to determine the effects of the intervention on the primary outcomes, comparing participants who attended at least half of intervention sessions to those who attended less than half. Logistic regressions were used for primary outcomes and linear and logistic regressions were used for secondary psychosocial outcomes, parallel to the main analyses described above.

## Cost and Cost-Effectiveness

Using standard methodologies to estimate cost and cost effectiveness [40, 41], we tracked costs of all activities associated with the intervention (excluding research related costs), including the cost of recruitment, client retention, session implementation, training, materials, and other general costs associated with the intervention for the first six online groups. These costs were reported in surveys by the study coordinator, intervention facilitators, and their supervisor weekly during the recruitment, training, and session implementation periods.

Categories of costs included administrative (e.g., entering attendance data, sending reminder texts, ordering supplies/materials, mailing materials); fidelity rating (e.g., listening to/rating session for fidelity, providing/receiving feedback in supervisor-facilitator sessions); gift card cost, distribution, and logging; pre/post group arrangements (e.g., meeting set-up, emailing materials); recruitment (e.g., community outreach, agency in-reach, social media promotion); session implementation (preparing for or facilitating sessions, providing referrals); training (on intervention manual/procedures); materials (e.g., printing/mailing materials); and other activities (not captured by the other categories). Fixed costs, which do not depend on the number of participants in a group, included training, session implementation, and fidelity. Variable costs included administrative activities, gift cards, materials, and recruitment.

The reported time spent on each activity was multiplied by a staff member’s respective hourly wage rate to determine total time costs. Due to the administrative burden required to accurately detail all costs, cost surveys were only completed for the first six online groups. Data from these six groups were used to estimate costs for the remaining groups in which data were incomplete or not collected. To estimate the total cost of intervention implementation for the cost-effectiveness analysis, we multiplied the average cost per group by 13 (as we conducted 13 groups).

We report the cost-effectiveness of the intervention as the average cost per person of a 10% increase in the self-reported rate of HIV testing. We estimated the increase in testing based on the percent difference in HIV testing rates between the treatment and control groups measured during the follow-up period. (Because the intervention did not have an effect on PrEP use, as described below, this analysis was only conducted for HIV testing.)

## Results

### Participant Flow and Characteristics

**Fig. 1:**
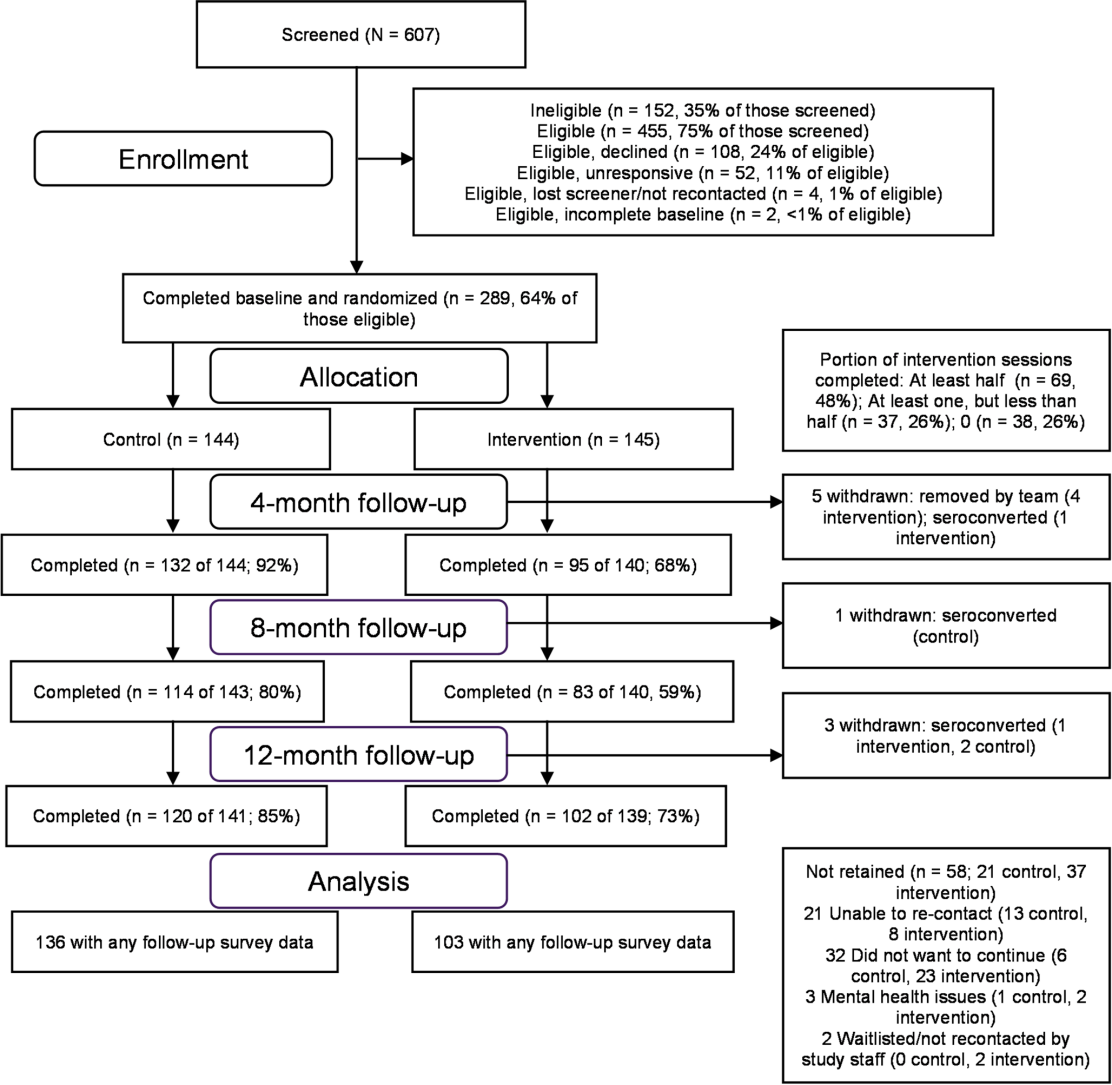
CONSORT diagram of participant flow in the *Siempre Seguiré* randomized controlled trial

As shown in Fig. 1, 607 individuals were screened between February 2021 and November 2023; a total of 455 (75%) were eligible, of whom 289 (64%) completed the baseline survey and were randomized (144 control, 145 intervention). Nine participants were withdrawn after baseline (and, thus, not included in the final retention rate): 5 (3 control, 2 intervention) were diagnosed with HIV, 1 moved out of the country, 1 no longer had reliable access to a cell phone to do study assessments, 1 was removed by the study team due to inappropriate behavior, and 1 was deceased. Fifty participants (who were not withdrawn) did not complete any follow-ups, ten only responded to the 4-month follow-up, two only responded to the 8-month follow-up, and five responded to both the 4- and 8-month (but not the 12-month) follow-ups. Of the 145 participants assigned to the intervention condition, 74% attended at least one intervention session (with 48% attending at least half). Among those who attended at least one intervention session, the mean number of sessions attended was 5 (SD = 3) for participants attending in-person sessions and 8 (SD = 4) for participants attending virtual sessions.

Table 1 shows the socio-demographic characteristics of the final baseline sample. The majority was from Mexico (61.6%), and over half (51.2%) were of undocumented immigration status; most (57.9%) had spent over 10 years in the U.S. Over three-fourths (78.5%) had a high school diploma or equivalent, and most (84.4%) were working full- or part-time. Of those who responded to the income question, about a third (36.2%) had incomes of at least $50,000 annually. Nearly all (99.6%) identified as gay or another sexual minority identity. Nearly 60% reported having an HIV test in the last 12 months, and 28.7% reported current PrEP use at baseline.

Of the socio-demographic variables, only length of time living in the U.S. and legal immigration status significantly differed by intervention condition at baseline (*p* <.05) and therefore were included as covariates in all intervention tests: On average, intervention participants had lived longer in the U.S. [mean (SD) = 3.3 (1.7) vs. 2.8 (1.8), t = 2.2, *p* =.03] and were more likely to have legal immigration status (53.1% vs. 40.6%, *p* =.04 per Fisher’s Exact Test) than were control participants.

### Primary HIV Testing and PrEP Outcomes

As shown in Table 2, intention-to-treat logistic regressions showed that intervention participants were more likely to get tested for HIV at follow-up than were control participants. This finding was significant for the overall sample and among participants who were not on PrEP at baseline. The intervention effect for PrEP use was not significant.

**Table 2:** *Siempre Seguiré* intervention effects on PrEP and HIV testing outcomes at Follow-up (among participants who completed any follow-up assessment)

	Intervention %	Control %	OR (95%CI), *p*-value
Any HIV Test at Follow-up, overall (%, *n* = 239)	93.2% (96/103)	83.8% (114/136)	2.5 (1.0–6.3.0.3), *p* =.049
Any HIV Test at Follow-up, among participants not on PrEP (%, *n* = 165)	90.1% (64/71)	76.6% (72/94)	2.6 (1.0–6.7.0.7), *p* =.04
Any PrEP Use at Follow-up (%, *n* = 239)	54.4% (56/103)	52.2% (71/136)	1.1 (0.6–1.8), *p* =.83

Note: All models controlled for immigration status and length of time in U.S

### Secondary Psychosocial Outcomes

As shown in Table 3, repeated-measures intervention efficacy tests indicated that at follow-up (vs. baseline), intervention participants experienced reduced anticipated internalized HIV stigma, internalized Latino stigma, and internalized sexual minority stigma, and showed lower agreement with PrEP-related stigmatizing beliefs than did control participants. Moreover, intervention participants showed lower medical mistrust on the general medical mistrust scale and marginally lower PrEP-specific conspiracy beliefs. No significant overall intervention effects emerged for other measures of coping or PrEP readiness.

**Table 3:** Effect of *Siempre Seguiré* on psychosocial outcomes (among participants who completed any follow-up assessment)

	Baseline M (SD) or %		4-Month M (SD) or %		8-Month M (SD) or %		12-Month M (SD) or %		b (SE) or OR (95% CI), *p*
	Intervention	Control	Intervention	Control	Intervention	Control	Intervention	Control	
**Stigma**									
Internalized sexual orientation stigma	1.90 (0.84)	2.02 (0.96)	1.64 (0.76)	1.89 (0.88)	1.67 (0.77)	1.79 (0.84)	1.61 (0.68)	1.87 (0.83)	− 0.14 (0.06), *p* =.02
Internalized Latino stigma	1.27 (0.45)	1.32 (0.45)	1.25 (0.42)	1.38 (0.46)	1.25 (0.45)	1.30 (0.40)	1.21 (0.41)	1.33 (0.45)	− 0.09 (0.04), *p* =.048
Anticipated internalized HIV stigma	3.16 (1.23)	3.01 (1.28)	2.91 (1.37)	3.12 (1.24)	3.03 (1.34)	3.14 (1.30)	2.82 (1.35)	2.91 (1.18)	− 0.22 (0.11), *p* =.048
Stigmatizing PrEP beliefs	2.47 (0.98)	2.43 (1.00)	2.07 (0.97)	2.42 (0.99)	2.08 (0.86)	2.28 (0.93)	2.05 (0.98)	2.32 (1.01)	− 0.33(0.09), *p* =.0006
**Medical Mistrust**									
PrEP conspiracy beliefs	2.36 (0.96)	2.31 (1.01)	2.07 (1.05)	2.20 (0.95)	2.14 (1.00)	2.33 (1.02)	2.02 (1.02)	2.14 (1.02)	− 0.17 (0.10), *p* =.07
General Medical Mistrust	2.84 (0.99)	3.14 (1.04)	2.71 (1.15)	2.96 (1.10)	2.71 (1.24)	2.94 (1.13)	2.40 (1.22)	2.81 (1.12)	− 0.22 (0.11), *p* =.048
**General Coping**									
Social Support Seeking	2.10 (0.85)	2.31 (0.91)	2.14 (0.96)	2.05 (0.89)	2.04 (0.84)	2.02 (0.91)	2.02 (0.92)	1.96 (0.92)	0.14 (0.09), *p* =.14
Cognitive/Reframing	2.95 (0.83)	2.99 (0.82)	2.66 (0.90)	2.51 (0.94)	2.35 (0.87)	2.38 (0.90)	2.44 (0.90)	2.37 (0.99)	0.07 (0.09), *p* =.45
Negative Strategies	1.73 (0.71)	1.82 (0.79)	1.62 (0.70)	1.65 (0.67)	1.64 (0.62)	1.63 (0.67)	1.54 (0.64)	1.49 (0.60)	0.03 (0.07), *p* =.64
**PrEP Readiness**									
Believe appropriate PrEP candidate	28 (48.3%)	45 (54.2%)	31 (59.6%)	41 (54.0%)	26 (56.5%)	41 (61.2%)	20 (41.7%)	31 (51.7%)	0.93 (0.50–1.73), *p* =.81
Have or know PrEP prescriber	17 (29.8%)	24 (29.3%)	30 (58.8%)	35 (47.3%)	28 (60.9%)	35 (52.2%)	31 (63.3%)	36 (59.0%)	1.23 (0.66–2.30), *p* =.52

Note: All models controlled for immigration status, length of time in U.S., and the baseline value of the outcome

### Within-Intervention Group Dosage Analysis

Intervention participants who attended at least half of the intervention sessions showed greater PrEP readiness at follow-up, including believing they were an appropriate PrEP candidate [OR (95%CI) = 3.0 (1.2–7.2), *p* =.02], and having or knowing a PrEP provider [OR (95%CI) = 3.8 (1.3–10.6), *p* =.01]. They also showed reduced PrEP-related stigmatizing beliefs [b (SE) = −0.33 (0.14), *p* =.03], reduced general medical mistrust [general: b (SE) = −0.34 (0.16), *p* =.04], and increased use of social support-seeking as a coping strategy [b (SE) = 0.25 (0.13), *p* =.05].

### Cost and Cost-Effectiveness

The cost per online group was $7,780 on average (range = $4,299 to $12,493). The total cost of 13 online groups would be $101,133, which equates to $982 per person (across 103 participants who attended intervention sessions; average number of participants attending sessions across groups = 12.1, SD = 3.4, range 6–17). The cost of the first two groups, when study staff were learning content and procedures, were highest. Costs were primarily driven by session implementation ($23,615, 23% of costs), administrative activities ($20,377, 20%) and fidelity ($19,449, 19%), followed by recruitment activities ($12,527, 13%) and gift cards ($11,427, 11%). Hard copy materials and supplies ($8,682, 9%), pre-session preparations and post-session activities ($3,531, 4%), training ($1,142, 1%), and other costs ($384, < 1%) accounted for smaller percentages of the total cost.

We estimated the increase in HIV testing based on the percent difference in HIV testing rates between the treatment and control groups measured during the follow-up period (9.4% percentage point difference, 11.2% percent increase over the control group), which resulted in a normalized cost-effectiveness of $876 per 10% increase in testing. Because fidelity and recruitment activities would likely be conducted much less intensively or not at all in community-based implementation, we completed a sensitivity analysis that excluded these costs—which indicated a drop in cost per person to $671, with a cost-effectiveness of $600 per 10% increase in testing. Variable costs were approximately $466 per person, implying that cost effectiveness could be improved by increasing the number of participants per group.

## Discussion

A randomized controlled trial of *Siempre Seguiré*, a community-based group CBT intervention, showed effects on increased HIV testing and reduced internalized stigma (for Latino and sexual minority identity, and regarding HIV) and medical mistrust among immigrant LGBMSM, using intention-to-treat analyses. Our results are aligned with intervention content, which used the CBT model to frame both internalized stigma and medical mistrust as responses to discrimination that may impede effective HIV prevention behaviors. Our results also are consistent with research demonstrating that CBT interventions can effectively reduce internalized stigma [16] and improve HIV prevention behaviors [15, 42, 43]. Moreover, our cost effectiveness estimates fall within the bounds of prior cost-effective behavioral HIV prevention interventions for gay, bisexual, and other men who have sex with men [44], although our sample size was too small to examine whether increased HIV testing rates in the intervention condition could identify a higher number of HIV diagnoses over time. Notably, pilot studies of *Siempre Seguiré* among people living with HIV indicated that the intervention increases ART adherence, improves coping (including decreased internalized stigma/shame) in response to discrimination, and reduces medical mistrust [18, 20].

Taken together, our body of work suggests that *Siempre Seguiré* is an evidence-based intervention that has the potential to improve overall health and well-being among Latino people affected by HIV and living with HIV, given prior research showing associations of improved coping and reduced stigma with mental and physical health [45–50], and HIV-related prevention and treatment outcomes [51–53]. However, with the exception of *Siempre Seguiré*, most anti-stigma interventions have addressed a single stigma (e.g., from HIV, sexual minority identity) and have not used an intersectional, holistic approach that takes into account the mix of levels and types of stigmas that LGBMSM individuals face [54].

*Siempre Seguiré* led to higher levels of HIV testing, but not greater PrEP use during the 12-month follow-up period. PrEP uptake increased in both the intervention and control groups over time. This finding may be due to increased access to PrEP navigation and services available for all participants through the partner community-based organization. Further, the intervention did not address structural barriers to PrEP use, such as access to a regular health care provider and health care insurance, especially for participants who did not have legal immigration status—underscoring the need for complementary structural interventions. The intervention participation rate also likely contributed to our lack of PrEP findings. As-treated, but not intention-to-treat effects, conducted only among participants who were exposed to the intervention, indicated significant effects on PrEP readiness. Although exposure to the intervention may have increased motivation to take PrEP, it is also possible that people who attended the intervention sessions were already more motivated to take PrEP than those who did not.

Limitations of study include the specific setting and population (a highly trusted community-based organization in Los Angeles County, California, that serves a majority-Mexican immigrant client base), which limits generalizability, as well as use of multiple different intervention facilitators across groups, which could have led to unmeasured variability in intervention delivery. In addition, the study was primarily conducted during the COVID-19 pandemic, a unique time globally that limited access to health care, including PrEP, and required mostly online data collection and intervention delivery. Individuals without digital access and those with low online literacy would not have been able to participate. We also experienced challenges in collecting objective data and, thus, were unable to validate self-reported PrEP use or adherence for the entire sample. Although we did collect objective HIV testing dates from medical records for much of the sample, unfortunately, the funder terminated the grant prematurely (based on a 2025 federal executive order) before these data could be processed fully. For the cost effectiveness analysis, we only collected data for a subsample of the groups, all of which were online. Thus, we could not account for in-person group costs, and our cost estimates only inform online program implementation. Our study, with its eight-session intervention, was designed prior to the publication of meta-analytic findings about HIV-related interventions showing that programs containing nine or more sessions had stronger effects on improved psychosocial outcomes [15]. Future tests of this or similar interventions could consider a longer treatment duration. Finally, although the intervention encouraged participants to be change agents in their communities, complementary structural-level interventions are needed.

In sum, our research demonstrated that *Siempre Seguiré* is effective in addressing intersectional stigma and increasing HIV testing among immigrant LGBMSM, an underserved population. Our results have implications for conducting culturally congruent HIV prevention interventions and for clinical practice. For example, when working with LGBMSM clients, counselors could use CBT strategies to identify, validate, and address internalized stigma and medical mistrust beliefs that may be barriers to adaptive coping—and, in so doing, promote the clients’ overall health and well-being. Many participants extolled the benefits of group participation with peers, i.e., other LGBMSM, outside of dating or sexual contexts, and the helpfulness of learning from each other in a facilitated manner. Thus, agencies might consider the creation of affinity spaces led by counseling staff so that the unique combination of LGBMSM identities can be expressed and celebrated alongside skill development. In addition, future research is needed to understand psychosocial mechanisms of action for intervention effects, as well as how to implement *Siempre Seguiré*. Specifically, research could be conducted to identify what aspects of *Siempre Seguiré* lead to, or do not lead to, behavior change, such as variations in intervention content and effects by facilitator characteristics, group dynamics, and client composition—and determine how key intervention components fit under current policy and funding streams. Such research would help to elucidate necessary active ingredients to implement, disseminate, and sustain behavioral HIV interventions such as *Siempre Seguiré* effectively.

## Data Availability

All authors ensure that the data support the published claims and comply with field standards.
